# Sensor Fusion and Advanced Controller for Connected and Automated Vehicles

**DOI:** 10.3390/s23167015

**Published:** 2023-08-08

**Authors:** Boyuan Li, Yafei Wang, Georgios Papaioannou, Haiping Du

**Affiliations:** 1 Research Centre for Intelligent Transportation, Zhejiang Lab, Hangzhou 311121, China; 2 Institute of Intelligent Vehicle, School of Mechanical Engineering, Shanghai Jiao Tong University, Shanghai 200240, China; wyfjlu@sjtu.edu.cn; 3 Intelligent Vehicles & Cognitive Robotics, Technische Universiteit Delft, 2600 AA Delft, The Netherlands; g.papaioannou@tudelft.nl; 4 Faculty of Engineering and Information Science, University of Wollongong, Wollongong 2522, Australia; hdu@uow.edu.au

Nowadays, intelligent vehicles are equipped with a number of advanced sensors, such as radar and cameras. The measurements of these additional sensors can be fused, and a sensor fusion system can be built based on various artificially intelligent algorithms. In this way, a large number of highly reliable measurements and estimates are available. This enriched measurement information can be greatly beneficial to the development of autonomous driving techniques, from perception and path planning to path tracking control layers (such as integrated chassis control). These autonomous driving methods can be applied to intelligent vehicles or intelligent vehicles in a connected vehicle platoon.

The Special Issue of *Sensors* aims at reporting on some of the recent innovative studies on the perception, decision-making, planning, and control layers of autonomous vehicles and vehicle platoons with the help of advanced sensor techniques. In the perception layer and state estimation layer, a traffic sign recognition method [1] was proposed, and a rail micro-crack detection method [2] was designed to improve the perception of small traffic targets and rail flaw detection. In addition, an efficient and high-precision estimation framework for a Four-Wheel Independently Actuated (FWIA) autonomous vehicle was focused on [3], and an efficient measurement method for brake pressure change rate was reported [4,5]. In the decision-making and path-planning layers, a decision-making framework based on the Extended Collision Warning System (ECWS) [6] and a collision relationship-based decision-making method based on the Deep Recurrent Q Network (CR-DRQN) [7] were proposed. Furthermore, a path planning method based on the Takagi–Sugeno (TS) fuzzy-model-based approach [8] and a motion planning method by minimizing motion sickness [9] were proposed. Some other studies focus on the vehicle control layer for electric vehicles, including a driving-adapt optimization strategy for a Magneto Rheological Fluid Transmission (MRFT) [10] and a robust speed tracking control strategy for an Integrated Motor-Transmission (IMT) powertrain system [11]. Another research study focuses on high-definition mapping, vehicle control, and Vehicle-to-Infrastructure (V2I) communication for the Robo-Taxi autonomous driving service, which covers the perception, V2I communication, and control layers of autonomous driving [12].

(1) Perception and state estimation layer

First, some new advanced measurement and estimation approaches based on advanced sensors are reported as follows:

Aiming at the challenges of small traffic signs, inconspicuous features, and low detection accuracy, a traffic sign recognition method based on the improved You Only Look Once v3 YOLOv3 was proposed [1]. Integrating the spatial pyramid pooling structure into the YOLOv3 network structure can realize the fusion of local features and global features, and a fourth feature prediction scale of 152 × 152 is applied to make full use of the shallow features in the network to predict small targets. Furthermore, bounding box regression is more robust when using a Distance-IoU (DIoU) loss by considering the distance between objects and anchors, the overlap rate, and the scale. By utilizing the equipped camera and advanced sensing technology, such as the data-driven sign recognition method, the detection network’s accuracy is substantially enhanced while the network’s real-time performance is kept as high as possible.

A rail microcrack detection method based on the magnetoacoustic coupling effect was proposed in [2]. The magnetoacoustic coupling detection technology is a nondestructive test that can detect early damage to the rail and achieve early warning of rail faults. In this study, the multi-physical field method, which includes electric fields, magnetic fields, and sound fields, is applied to achieve real-time detection of the rail crack. As the excitation source, the pulse electrical signal is easily received by the sensors, and the control of the pulse electric signal source is very flexible. In addition, through the adjustable magnetic field space and the focusing detection of the magnetoacoustic signal receiver, ultrasonic detection resolution can be realized. Furthermore, this detection method combined magnetic acoustic detection with spectrum analysis to obtain a higher-resolution image than the frequency of the magneto-acoustic signal receiver. Through the sensors of the magnetic signal and acoustic signal, this method has great feasibility and development potential in the field of rail flaw detection.

An efficient and high-precision estimation framework for Four-Wheel Independently Actuated (FWIA) autonomous vehicles was proposed based on a novel tire model and an Adaptive Square-root Cubature Kalman Filter (ASCKF) estimation strategy [3]. First, a reliable and concise tire model that considers the tire’s nonlinear mechanics characteristics under combined complex conditions is proposed. Through the Piecewise Affine (PWA) identification method and based on the experimental tire data, this tire model can improve the lateral dynamics model of FWIA. An ASCKF algorithm based on the Maximum A Posteriori (MAP) criterion is proposed in the vehicle state estimation framework, which includes the estimation of longitudinal force, yaw rate, and sideslip angle based on easily measured signals (steering wheel angle, wheel angular velocity, longitudinal acceleration, and lateral acceleration). Through the co-simulation of CarSim and Simulink, the proposed ASCKF estimation algorithm can still maintain high accuracy and stability when the state changes suddenly or the statistical characteristics of noise are unknown.

The studies on the measurement method of Pressure Change Rate (PCR) were proposed in [4,5]. First, an efficient measurement method for brake pressure change rate is proposed based on Poiseuille’s law. Then a new measurement device, including the core components of an isothermal container and a laminar flow resistance tube, is designed. The design parameters of an isothermal container are optimized through thermal insulation performance tests, flow resistance tests, and measurement accuracy tests. Furthermore, the core parameters, such as the number, radius, and length of laminar flow channels for the laminar flow resistance tube, are analyzed and optimized. The simulation model of the PCR test system for the commercial vehicle brake chambers is presented, and the hardware system of the PCR experimental test is designed based on an iosthermal vessel pressure sensor and a differential pressure sensor. Through the comparison of the measurement results of simulation and experiment, the proposed PCR measurement method is verified.

(2) Decision-making and path-planning layers

Based on advanced estimation and sensor technology, the research studies about the decision-making, path planning, and control layers of autonomous vehicles can be reported as follows:

In [6], based on the concept of a Product-Service-System (PSS), an Extended Collision-Warning System (ECWS) was modeled as a smart PSS. A comprehensive process for developing a smart PSS conceptual model is presented, and an integrated ECWS PSS solution including intelligent products and warning services is designed based on the Theory of Inventive Problem Solving (TRIZ). The major contribution of this research includes several aspects: (1) In the aspect of PSS research, a stepwise method to create a smart PSS conceptual design is presented; (2) In the aspect of CWS research, first a novel understanding and conceptual framework of ECWS from the perspective of PSS are presented. Then an ECWS modeling approach is designed by integrating the intelligent product system, stakeholders, and collision warning service system module. The designed ECWS can assist drivers and pedestrians by avoiding vehicle collisions and vehicle-pedestrian collisions.

In the study [7], a collision relationship-based driving behavior decision making method for intelligent land vehicles was presented, which successfully solved the problem of instability in decision-making caused by decreased perceptual confidence. This method is based on the approach of the Deep Recurrent Q Network (DRQN), which is a combination of the Deep Q Network (DQN) and the Long-Short Term Memory (LSTM). LTSM is a group of networks with loops in them that retain memory about the previous state. The major contribution of this study includes the following aspects: (1) The collision relationship between an intelligent vehicle and surrounding vehicles is utilized as the state input rather than the positions and velocities of all the vehicles. (2) By utilizing the LSTM to train the time-series input, the proposed method effectively weakens the adverse effects of reduced perception confidence; (3) The experiments verify that the proposed algorithm is superior to traditional DQN.

In [8], instead of a linear vehicle model with the cost of lost accuracy, a Takagi–Sugeno (TS) fuzzy-model-based closed-loop Rapidly Exploring Random Tree (RRT) algorithm with an online re-planning process was applied to build the motion planner. This method effectively improves the vehicle’s performance in dynamic obstacle avoidance while greatly reducing the computational effort and planning the local obstacle avoidance path in line with the dynamic characteristics of the vehicle. More specifically, the TS-RRT planner is integrated with a low-order controller to expand the RRT and conduct the online re-planning process by considering the close-loop dynamics. The TS-RRT planner algorithm uses controller outputs and the T-S fuzzy dynamics model to conduct a forward simulation process, and the predicted trajectory is calculated and its feasibility checked based on the vehicle and environment boundary, including rollover and obstacle avoidance constraints. The numerical simulation results prove that the proposed motion planner can effectively generate a reference trajectory that guarantees driving efficiency with a lower re-planning rate.

The application of motion planning in order to minimize motion sickness in automated vehicles was presented in [9]. The aim of the path planning solution is to ensure the optimum compromise between motion comfort, safety, driving behavior, energy efficiency, journey time, and riding confidence. In this direction, an optimal control problem is formulated through which we seek the optimum velocity profile for a predefined road path for multiple fixed journey time (JT) solutions. This optimal control is focused on motion sickness minimization and a sorting algorithm is applied to seek the optimum solution among the pareto alternatives. In the performance metrics, comfort, driving behavior, energy efficiency, vehicle stability, and subjective feel-oriented objectives are considered. In the motion comfort-oriented metrics, whole body vibration and illness rating are applied to present comfort and motion sickness. The aggressive driving metrics include the Root Mean Square (RMS) values of longitudinal and lateral jerk. The energy efficiency-oriented metrics refer to the total energy demand, which can be calculated as the product of the instantaneous force produced by the propulsion motor and the velocity of the vehicle. In the vehicle stability-oriented metrics, suspension travel and rollover stability are used. The metric that considers the subject feel of passengers, which is called the perceptible roll index, is designed to combine the roll gradient and the position of the passengers.

(3) Vehicle control layer

A driving-adapt strategy was proposed for the electric vehicle equipped with Magneto-Rheological Fluid Transmission (MRFT) in [10]. In this study, in order to fully explore the driving-adaptive potential of electrical vehicles, the vehicle in this study is equipped with the MRFT. First, for the purpose of the analysis of the quantitative relationship between the driving condition and the powertrain system, the powertrain model considering the energy transmission process and the driving-adapt transmission model considering MRFT are proposed. Secondly, a driving-adapt optimization strategy in the specific driving condition is designed by considering two steps: (1) choosing the MRFT fixed speed ratio and the relevant MRF parameters; and (2) solving the relevant control variables of the powertrain in the driving conditions. Finally, the results prove that the proposed driving-adapt strategy for electric vehicles equipped with MRFT has the potential to alleviate the problem caused by the incompatibility of the powertrain characteristics and driving conditions.

In [11], a robust speed tracking controller for future electric vehicles equipped with an integrated motor-transmission (IMT) powertrain system was designed. The speed tracking controller becomes challenging due to the time delay of the in-vehicle signal transmission, unknown slope variance, system parameter uncertainty, and signal measurement noise. In order to address the above issues, first a disturbance observer and low-pass filter are developed to improve the measurement and estimation performance. Then a network-induced delay speed tracking model is proposed by considering the damping coefficient uncertainties of the IMT powertrain system. After that, a novel Lyapunov function is proposed to design the robust speed tracking controller using the linear matrix inequality (LMI) algorithm. The simulation results demonstrated that the proposed method has strong robustness, excellent speed tracking performance, and ride comfort.

An autonomous driving software stack was proposed for the main mission of the robot-taxi service on public roads [12]. Among the key requirements to achieve this mission, this study is focusing on high-definition mapping, vehicle control, and Vehicle-to-Infrastructure (V2I) communication. The main potential contributions of this paper include providing practical procedures for developing an HD map, a longitudinal speed controller, and the services implemented by V2I technology. Particularly, an HD map for use in prior map-based localization is proposed. Then a disturbance observer (DOB)-based longitudinal speed controller is designed and presented. Finally, the development of V2I communication enabled the Traffic Light Recognizer (TLR) to achieve a nearly perfect recognition rate. Furthermore, the robo-taxi service handler was developed to perform the main mission of the contest.
